# Leiomyoma of the Female Urethra—A Rare Tumor: Case Report and Review of the Literature

**DOI:** 10.1155/2012/280816

**Published:** 2012-07-15

**Authors:** Mrinal Pahwa, Yusuf Saifee, Archna R. Pahwa, Manu Gupta

**Affiliations:** Department of Urology, Sir Ganga Ram Hospital, New Delhi 110060, India

## Abstract

Leiomyoma is a benign smooth muscle tumor which is rarely found in urethra. Only a handful of cases have been reported in the literature. We hereby report a case of urethral leiomyoma in a twenty-seven-year-old female who presented with intermittent hematuria. Mass was completely excised with a rim of normal tissue. Patient remained asymptomatic with no evidence of recurrence in followup.

## 1. Introduction

Urethral leiomyomas are rare benign tumors arising from the smooth muscle of urethra. These are more common in women [1]. Approximately 40 odd cases have been reported in world literature [1–9].

## 2. Case Report

**Figure 1:**
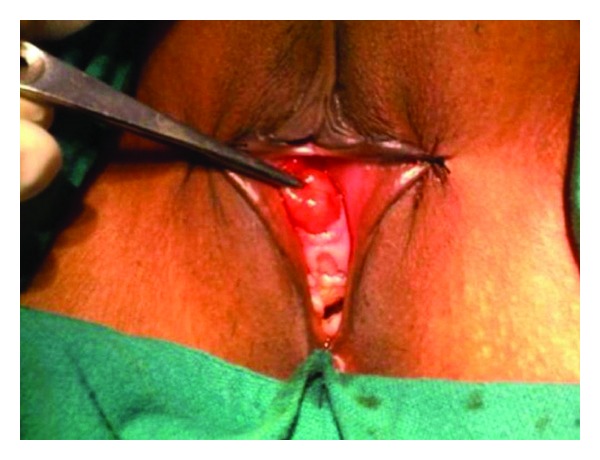
Local examination revealed a 2  ×  2 cm growth arising from urethral meatus.

**Figure 2:**
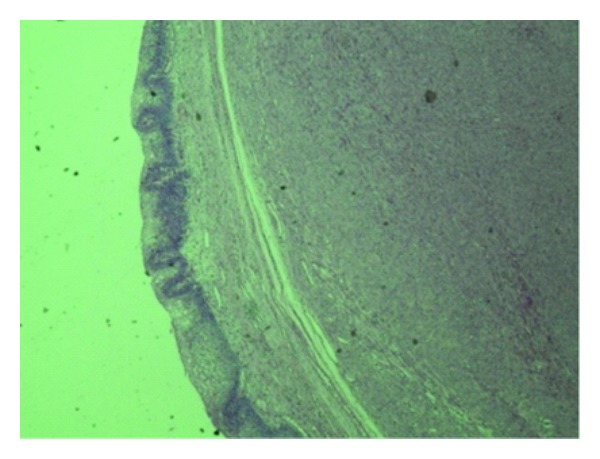
Histopathology 40x-bladder mucosa with submucosal encapsulated leiomyoma.

 A twenty-seven-year-old woman presented with intermittent hematuria and a mass coming out from the vagina. On examination, she was found to have a fleshy mass at the urethral meatus (Figure 1). She did not have any voiding symptoms or perineal pain. On urethroscopy, the mass was occupying the mid and distal urethra from 10 o′clock to 6 o′clock position. Cystoscopy was normal. The urethra could be calibrated up to 18 Fr. The mass was excised and the proximal urethral mucosa was mobilized and approximated with the distal edge. After excision of the tumor, the urethra could admit a 30 Fr dilator. Patient was catheterized for 48 hours and voided well on catheter removal. Histopathological examination revealed an encapsulated tumor composed of spindle-shaped smooth muscle fibers arranged in a whorling pattern. On immunohistochemical examination tumor was positive for vimentin and smooth muscle actin. A diagnosis of leiomyoma was made (Figure 2).

## 3. Discussion

Leiomyoma of urethra is a rare condition affecting women more than men. The tumor is most common in third and fourth decade; the mean age of the appearance is around 41 years [8, 9]. Distal urethra can be affected but proximal segment is the most common site [3]. Common presenting symptoms include urinary tract infection (64.3%), a mass (50%), dyspareunia, urinary retention, and irritative lower urinary tract symptoms. The tumor has been reported to enlarge during pregnancy and shrink after delivery, suggesting a possible hormonal dependence [5].

A differential diagnosis of urethrocele, a urethral diverticulum, caruncle, and malignancy should be considered. A careful clinical examination, urethroscopy, and radiological examination of the lower urinary tract are essential to distinguish it from urethrocele, urethral diverticulum, and caruncle. However, a microscopic examination is indispensable to exclude possibility of a malignancy although lately transvaginal ultrasound and MRI have been used to indicate the benign nature of the disease [10].

Urethral leiomyomas must also be differentiated from paraurethral leiomyomas, which may be removed without disrupting the urethral muscle or mucosa. No malignant transformation or recurrence has been reported. Local surgical excision is the treatment of choice for urethral leiomyoma [6].

Our case was different as the patient presented with episodic hematuria unlike the previously reported cases. The mass was located in distal urethra which is not the common site of presentation of leiomyoma in females.
